# Cellular and Molecular Evidence of Multiple Sclerosis Diagnosis and Treatment Challenges

**DOI:** 10.3390/jcm12134274

**Published:** 2023-06-26

**Authors:** Zuber Khan, Ghanshyam Das Gupta, Sidharth Mehan

**Affiliations:** 1 Division of Neuroscience, Department of Pharmacology, ISF College of Pharmacy, IK Gujral Punjab Technical University, Jalandhar 144603, India; zuber@isfcp.org; 2 Department of Pharmaceutics, ISF College of Pharmacy, IK Gujral Punjab Technical University, Jalandhar 144603, India; drgdg@rediffmail.com

**Keywords:** multiple sclerosis, neurodegeneration, autoimmune reactions, biomarkers, disease-modifying agents

## Abstract

Multiple sclerosis (MS) is a chronic autoimmune disease that impacts the central nervous system and can result in disability. Although the prevalence of MS has increased in India, diagnosis and treatment continue to be difficult due to several factors. The present study examines the difficulties in detecting and treating multiple sclerosis in India. A lack of MS knowledge among healthcare professionals and the general public, which delays diagnosis and treatment, is one of the significant issues. Inadequate numbers of neurologists and professionals with knowledge of MS management also exacerbate the situation. In addition, MS medications are expensive and not covered by insurance, making them inaccessible to most patients. Due to the absence of established treatment protocols and standards for MS care, India’s treatment techniques vary. In addition, India’s population diversity poses unique challenges regarding genetic variations, cellular and molecular abnormalities, and the potential for differing treatment responses. MS is more difficult to accurately diagnose and monitor due to a lack of specialized medical supplies and diagnostic instruments. Improved awareness and education among healthcare professionals and the general public, as well as the development of standardized treatment regimens and increased investment in MS research and infrastructure, are required to address these issues. By addressing these issues, it is anticipated that MS diagnosis and treatment in India will improve, leading to better outcomes for those affected by this chronic condition.

## 1. Introduction

MS is a demyelinating, inflammatory neurodegenerative disease that affects both the central and peripheral nervous systems, resulting in severe disability and motor neuron death [1]. It is the most common cause of non-traumatic neurological disability in children and adolescents. This motor neuron disease is most commonly diagnosed between the ages of 20 and 40, with women being diagnosed three times more frequently than men [2,3,4].

The number of people affected by MS worldwide increased from 2.3 million in 2013 to 2.8 million by 2020. MS International Federations, UK, has just released a new report in the Atlas of MS, third edition, indicating that the global prevalence of MS is estimated to be 36 peopleper 100,000 people, implying that 2.8 million people worldwide suffer from and live with MS [5]. There are low, intermediate, and high prevalence areas worldwide with varying MS incidence and prevalence rates [6]. Over the last three decades, the global prevalence of MS has increased by 10% every five years [7,8]. Every five minutes, someone aroundthe globe receives a diagnosis of this illness. MS affects not only adults but at least 30,000 people under 18, a figure significantly more significant than that reported in 2013 [7,9].

India is a vast, developing South Asian country with a diverse physical landscape, religion, customs, castes, races, and languages. This characteristic makes India a promising target for studying the epidemiology of various disorders, such as MS [10]. In the 1980s, it was estimated that approximately one in every 100,000 Indians had multiple sclerosis. According to data from hospitals across India, the annual diagnostic rate for MS patients has nearly doubled. In India, there has not been much investigation into the prevalence and incidence of MS. Singhal et al. estimated that 1.33 out of every 100,000 people had MS in an earlier investigation [11]. Based on these prior findings, we explore the therapeutic challenges connected with specific diagnostic biomarkers and an appropriate treatment plan for MS disease, which is becoming more prevalent in the Indian population and must be prevented from worsening. We’ve also investigated the causes of this disease’s recurrence and added a follow-up to the standard treatment. 

## 2. MS Epidemiological Status

MS was initially identified in India between 1954 and 1961 [12]. Due to the regional factors that may affect illness prevalence, it was widely assumed that MS was primarily a Western disease. Geographically, the average incidence is 4.15 in northwest India (above 15° N latitude) and 3.2 in south India (below 15° N latitude) [13]. On the other hand, the global distribution of MS shows that northern latitudes have the highest frequency [14], whereas Asian and Hispanic populations have a significantly lower prevalence. However, the prevalence of MS among African Americans is rising, implying environmental and genetic changes [15]. According to a retrospective cohort study that used electronic health records from the Kaiser Permanente plan in Southern California, African Americans had a 47% higher risk of developingMS. In contrast, Hispanic and Asian Americans had 50% lower and 80% lower risks, respectively (2.9 per 100,000 for Hispanics vs. 6.9 for whites) [15,16]. According to the latest study conducted in the US, white people were more likely to have MS than black people, people from other non-Hispanic racial and ethnic groupings, and Hispanic people. Even after accounting for race, ethnicity, age, and sex, the prevalence of MS rises noticeably and unevenly with latitude in the US. Across racial and ethnic groupings, the northern regions of the United States continue to have a greater frequency of MS [17].

The MS Society of India assists about 10,000 MS sufferers across India (Delhi, Kolkata, Pune, Chennai, Indore, and Hyderabad). MS was considered rare in India until the mid-1970s; now, with more neurologist updates of old diagnostic criteria, improved illness awareness, and breakthroughs in diagnostic equipment, it is being diagnosed more frequently [14]. The first attempt to assess the MS prevalence rate in India was made in the West Coast regions between 1975 and 1985. According to hospital statistics, the rate ranged between 0.17 and 1.33 per 100,000 [18]. According to a hospital-based study in India, MS-related neurology admissions increased from 1.8 to 2.54% in the recent decade [19]. MS International Federation World data forecast a prevalence of 3.99 per 100,000, substantially tripling prior estimates [20]. In comparison to India, the Middle East and North Africa region is in the low-to-moderate MS prevalence zone, with rates of MSincidence that are significantly higher than those in sub-Saharan Africa but slightly lower than those in Southern Europe. However, over the past few decades, there has been an apparent shift towards higher MS prevalence, consistent with the disease’s diversifying prevalence worldwide [18].Prevalence rates today range between 55 and 85 per 100,000 people, according to more recent data from Kuwait, Qatar, Bahrain, and the United Arab Emirates, which also showed an additional increase [21,22,23,24,25]. Misdiagnosis of MS has increased the disease’s prevalence and progression rate in India, where identical diagnostic criteria have been employed to confirm other neurodegenerative illnesses [10].

According to the MS international federations, the overall number of people with MS in India is around 145,800, with a prevalence rate of 11 per 100,000 [18]. Due to the increase in MS patients, there is a higher need for appropriate diagnoses and effective treatments for this motor neuron dysfunction [26]. In India, there has been little study on MS; no particular curative medication or molecular biomarkers are available to diagnose and treat the condition. All available treatments are only symptomatic and do not change the course of India’s illness [18,27]. As a result, there is no information about pre-clinical and clinical research data related to biomarkers and test medicines fortreating MS [28].

There has been substantial progress in understanding and treating MS in recent years [29]. To be diagnosed, the white matter of the central nervous system must exhibit lesions scattered across time and distance, and all other diagnostic alternatives must be ruled out [30]. The Schumacher clinical criteria were used to diagnose MS for the first time in India in the late 1950s. Since the 1980s, diagnostic tests worldwide, including in India, have been replaced by McDonald’s standards, including MRI, which has recently been updated to aid early diagnosis [18]. MS manifests clinically in India in the same way as it does elsewhere. The condition affects more women than men, and the average age at onset is between 25 and 30 years old (mean 27 years) [18]. Relapsing-remitting multiple sclerosis (RRMS), secondary progressive multiple sclerosis (SPMS), and primary progressive multiple sclerosis (PPMS) patterns are similar to those seen in the West [31]. 

Many MS diagnostic tests are routinely used in clinical practice in India. The diagnostic pathways for MS in India generally follow international guidelines, which include several diagnostic measures. Doctors first take a detailed medical history and physical examination to check for signs of neurological dysfunction [32]. The MRI test of the brain and spinal cord is a critical diagnostic tool used to detect MS lesions. Gadolinium-enhanced MRI is often used to detect active inflammation [14 in the main reference]. Another diagnostic test, the lumbar puncture test, is performedin which CSF analysis helps diagnose MS by detecting the presence of oligoclonal bands, which are abnormal antibodies produced in response to inflammation [33]. Another significant test used is the visual evoked potential, which measures the electrical activity of the visual pathways in response to visual stimuli. It helps diagnose MS when the patient has symptoms of optic neuritis. In conclusion, early diagnosis and treatment can help manage the symptoms and improve the quality of life of MS patients [34].

In India, both injectable and oral disease-modifying treatments are available. During an acute attack, methylprednisolone is given intravenously for 3–5 days. In RRMS treatment, disease-modifying therapy (DMT) is used to prevent relapses and impairment, and early treatment should be initiated to end all evidence of disease activity (NEDA). The patient should know the medication’s advantages, disadvantages, and costs. Many Indian patients are denied this benefit due to itshigh cost [35]. Aside from that, most treatment is symptomatic and supportive, especially for fatigue, gait difficulty, and sphincter disturbance, and there is currently no treatment for SPMS/PPMS [36]. There is no cure for MS, but several treatment options are available, and they are routinely used in India to manage the symptoms and slow down the progression of the disease. Disease-Modifying Therapies (DMTs) are medications that can reduce the frequency and severity of MS attacks. It includes interferon beta, glatiramer acetate, and fingolimod[101 main reference, Gajofatto]. Symptomatic treatment can also help manage MS symptoms such as muscle stiffness, fatigue, and bladder problems. Medications like baclofen, amantadine, and modafinil may be used [37]. Rehabilitation is also used in routine practice,which can help improve the patient’s mobility, coordination, and strength. Physical, occupational, and speech therapy may be used [38].

The outcome of MS is unpredictable, and there are currently no biomarkers available to predict the progression of a specific MS patient. Jena et al.’s clinical experience from a short, retrospectively investigated series supports the hypothesis that those with multiple symptoms and those with motor weakness, sphincter disruption, ataxia, and partial recovery will do poorly [36]. Increased awareness, substantial epidemiological research, specialist MS clinics, first-rate rehabilitation facilities, and cost-effective DMA are urgently needed in India [35,39]. The majority of patients at India’s public and teaching hospitals come from lower socioeconomic backgrounds, and gender disparities have a significant impact on data collection and analysis in these settings [40]. The greater availability of MRI equipment and better-trained neurologists, particularly in urban areas, could explain the increase in instances. Knowing these significant findings, we can conclude that India needs to urgently address the many issues associated with motor neuron diseases like MS to diagnose and treat the disease before it worsens.

**Figure 1:**
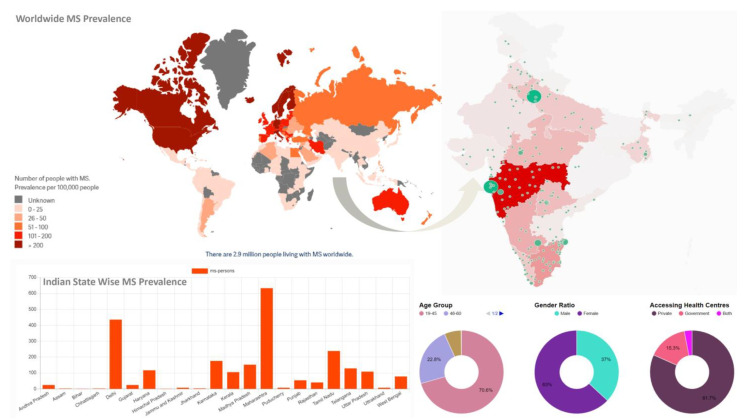
**Global MS incident and that of India showing per 100,000 prevalence rate**-[MS International Federation. Atlas of MS, 2023; https://www.atlasofms.org/; (access date 6 June 2023) India MS Map; https://indiamsmap.org/].

According to the data, the global population of people with MS has risen from 2.3 million in 2013 to 2.8 million in 2020 and 2.9 million in 2023. It emphasizes the numerous challenges and disparities that people with MS experience while seeking a diagnosis, treatment, or care [41]. According to the Multiple Sclerosis Society of India (MSSI) Indian Map of MS, the prevalence rate is 11 people per one hundred thousand. In India, the highest prevalence rate is in the states of Maharashtra and Delhi. The majority of people suffering from MS are females in the age range of 19–45. The private health centers of India have higher access to MS patients to diagnose and treat them than the government centers [42] (Figure 1).

## 3. AetiologicalFactors Responsible for MS 

The etiology of MS is complex and varied. Although it is commonly stated that the cause of MS is unknown, this is not entirely correct. Smoking, EBV, ultraviolet B (UVB), and vitamin D all play essential roles in the causal pathway that leads to MS development [43,44]. Immune mechanisms that occur across the white matter, such as neural inflammation, demyelination, remyelination, neurodegeneration, and glial scar formation, have been revealed to play an essential rolein the pathophysiology of MS [45].

### 3.1. Epstein-Barr Virus (EBV)

A virus may have produced immune-mediated demyelination in the brain and spinal cord [46]. The EBV is the top suspect among the probable causal agents. This human herpes virus remains dormant in B cells for the host’s life after infection [47]. The increased incidence of MS after infectious mononucleosis suggests that EBV has an underlying causal role [48]. Since the virus is not always present in MS lesions, identifying the precise mechanisms by which it contributes to the onset of MS remains challenging [49]. 

A prominent cause of EBV sickness is a breakdown in the immune system’s usual ability to regulate EBV infection. Even though EBV enters and changes B cells, the relationship between EBV and its host cells is also thought to be a possible cause of immunological failure [43]. According to a single study, EBV can produce inflammation in both the peripheral and central nervous systems, leading to a CNS lesion in peoplewith multiple sclerosis. Immune-evading EBV features and immunological deficiencies linked to risk promote inflammatory cascades in the periphery [43,50]. Based on this essential data, we can conclude that EBV is significantly associated with an elevated risk of MS.

### 3.2. Vitamin Ddeficiency

MS has been reported to be more common in nations far from the equator, suggesting that a lack of sunlight and low vitamin D levels may play an essential role in disease progression [51]. Although it is unknown whether vitamin D supplements can help with MS symptoms, its deficiency puts an MS patient at a higher risk of relapse since it is hypothesized to have a preventative effect on the development of MS [52]. Vitamin D deficiency is common in India, and it is difficult to identify its function in MS [53]. 

However, Pandit et al. found that vitamin D deficiency has an inverse relationship with MS, with people with low vitamin D levels having a higher incidence of MS and a higher risk of recurrence [54]. Two studies conducted in the USA found that elevated vitamin D levels have been linked to a lower risk of MS disease and lower clinical activity established in the disease, such as reduced disease activity on brain MRI and a lower chance of relapse [55,56]. Another study conducted in Switzerland discovered that Low serum Vitamin D levels significantly contribute to MS disease development [57]. Another American study found a link between MS disease and low vitamin D levels in childhood. Furthermore, it was shown that vitamin D has immunoregulatory capabilities in both adaptive and innate immunity, and its receptors are found on many immune cells such as astrocytes, oligodendrocytes, and microglia, having a significant impact on the development and function of the CNS. Its low level changes the CNS, promoting inflammation and further MS progression [58,59]. One more study in Saudi Arabia showed that vitamin D deficiency is a risk factor for MS, despite the absence of direct evidence for vitamin D’s effects on MS progression [60].

We might conclude from these significant findings that vitamin D plays an essential role in the etiology of multiple sclerosis. Vitamin D-rich foods in the diet and adequate daily sunshine exposure may help prevent the advancement of such a neurodegenerative condition.

### 3.3. Genes

MS is not directly inherited, but those with a family member who has it are more likely to develop it. A Class II human leukocyte antigen (HLA) gene is the most common susceptibility allele. More than 200 non-HLA single-nucleotide polymorphisms (SNPs) and at least one protective HLA allele have been discovered through advanced genomic platforms, advancing our understanding of the genetic code [61]. Numerous non-HLA SNPs are located near innate or adaptive immunity genes, indicating that MS is an immunological homeostasis disorder. Thus far, all the SNPs discovered are common gene variations (not disease genes) [39]. Common genetic variants contribute approximately 20% of heritability risk, while rare and low-frequency coding differences contribute approximately 5% [62]. 

Twin and familial clustering studies revealed that MS has a genetic component. Clinical concordance rates were found to be much higher in the former group (25–30%) than in the latter (3–7%) [63]. This finding may explain why MS has limited penetration or why any given genotype is more likely to develop the disease [64]. Half-siblings are less likely to acquire MS than full-siblings. On the other hand, people adopted by families with MS have a risk comparable to the general population. If both parents have MS, the risk is significantly increased. As a result of this factual data, we can conclude that genetic distribution among common variations is potentially connected with an increased risk of MS.

### 3.4. Tobacco Use

According to Briggs et al., peoplewho smoke have approximately twice the risk of developing multiple sclerosis as those who do not smoke [65]. Many studies have been published on the relationship between smoking and susceptibility to MS, and almost all have found a significant negative impact [66,67]. Cigarette smoke has a cellular effect on the immune system, causing it to release cytokines that cause inflammation. C-reactive protein, fibrinogen, and other inflammatory markers are higher among smokers, as are pro-inflammatory cytokines such as IL-6, IL-23, IL-1β, TNF-α, and IFN-γ [68,69]. 

Tobacco contains a lot of free radicals. According to findings, oxidative stress caused by free radicals causes genetic changes and plays a role in various neurological illnesses, including Parkinson’s disease and MS [68,70]. According to a particular study, smoking affects MS regardless of the age of exposure, and its detrimental effect gradually fades following smoking cessation [71].These significant findings show that smoking causes the release of inflammatory cytokines, which are strongly linked to an elevated risk of neurodegenerative illnesses like MS.

### 3.5. Adolescent Obesity

Obesity is increasingly prevalent worldwide, posing a severe public health problem [72]. Researchers discovered that compared to women with a normal BMI (18.5 to 21 kg/m^2^) at age 18, those with a BMI of 30 kg/m^2^ had a 2.25-fold higher chance of developing MS [73]. Childhood obesity and female hormonal factors, or the X-chromosome, may be causing the rising female-to-male ratio in MS, as evidenced by a higher risk of MS/CIS in moderately and very obese girls but not boys [72]. 

Obesity causes a considerable change in the number of Th1 and Th2 cells, with a drop in Th2 and an increase in Th1, comparable to the scenario seen in MS. Obesity increases Th17, another immunological marker examined in the context of MS [74]. Upregulation of CD8 T cells is another major shift in obesity that explains insulin resistance, which has recently been linked to MS [75]. These findings give solid evidence that obesity is significantly associated with the course of neurodegenerative illnesses such as MS.

### 3.6. Females Are More Susceptible to MS

Females are shown to be two to three times more likely than men to get MS, although the cause is uncertain [76]. According to one study, women were more likely to be exposed to environmental factors that rendered them more vulnerable to MS [77]. Women have a higher risk of developing clinically definitive MS (CDMS) after a first demyelinating episode, including optic neuritis, as seen in the historic Optic Neuritis Treatment Trial (ONTT); additionally, sex-specific reproductive exposure after clinically isolated syndrome (CIS), such as pregnancy, may increase the risk of CDMS [78,79]. This information suggests that women have a higher natural risk of developing MS than men.

### 3.7. Immune Responses 

**Figure 2:**
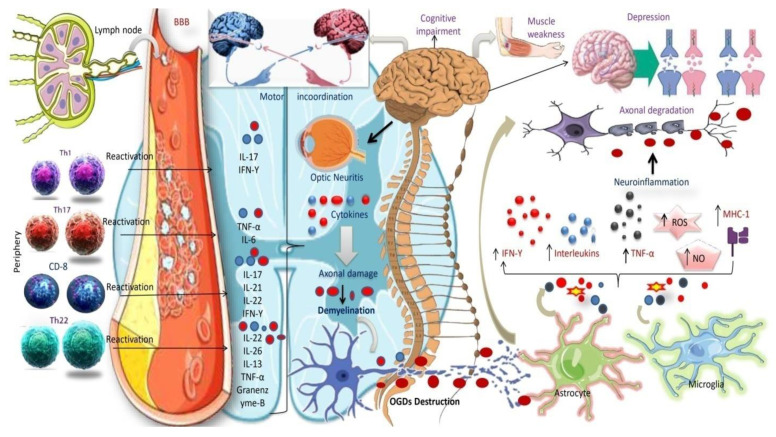
T-cells, cytokines, and non-neuronal cell overexpression in MS pathogenesis.

T lymphocyte cells invade the CNS via the vasculature when activated, killing the myelin sheath, nerve fibers, and Schwann cells. T-cell activation also activates B cells, causing them to go to the site of inflammation and release myelin-damaging antibodies [80]. Once CNS tissue is damaged, local immune cells, particularly microglia cells, become active. Other immune cells, such as CD4+ and CD8+ T cells, B cells, monocytes, macrophages, and dendritic-like cells, can enter CNS lesions quickly by up-regulating MHC classes I and II and cell surface co-stimulatory molecules, as well as secreting cytokines and chemokines [81] (Figure 2).

**Figure 3:**
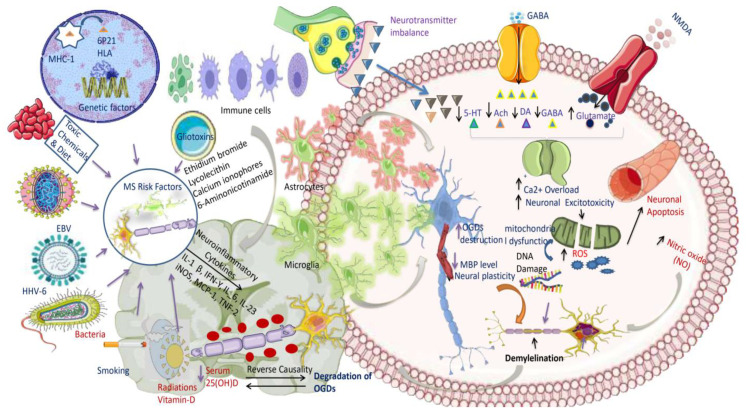
MS risk factor pathways and neurotransmitter imbalances leading to neurodegenerative illness in MS patients.

According to a study, MS may be caused by Th1 cells, which release IFN-γ, and Th17 cells, which release IL-17A [82]. However, anti-inflammatory Th2 cells may be able to counteract these pro-inflammatory responses. In MS patients with CSF and active MS lesions, IFN-γ and IL-17 transcripts and protein products have been observed [83,84,85,86]. IFN-γ producing T cells have been identified more often in the blood of MS patients shortly before acute attacks [27], and IFN therapy causes MS relapses, indicating that Th1 cells are implicated in the disease [87]. Based on these exciting results, we can assume that immunological reactions are critical in degrading the myelin sheath and oligodendrocyte damage, eventually leading to the neurodegenerative condition seen in MS (Figure 3).

The illustration depicts the activation of the Pathogenic T-helper (Th) cell fraction and the consequent release of inflammatory cytokines. Similar toTh17 subsets, self-reactive Th1, Th22, and Th1 are activated in peripheral lymph nodes, cross the BBB, and move to the CNS, detecting myelin as a foreign molecule and triggering an immune response.T-cells are reactivated in the CNS and, by secreting lineage-defining cytokines, regulate the functions of CNS resident cells (microglia, astrocytes, and OGDs) by increasing inflammatory cytokine production, antigen-presenting cell (APC) function, and apoptosis, thereby contributing to axonal damage and demyelination. Cytokines are tiny signaling molecules released by immune cells that are important in immune response regulation. Interferon-gamma (IFN-), tumor necrosis factor-alpha (TNF-α), and interleukin-17 (IL-17) cytokines are increased in MS and contribute to the inflammatory response in the CNS. These cytokines increase the formation of reactive oxygen species (ROS) and nitric oxide (NO), which can induce myelin and axon damage. Astrocytes in MS activate and release cytokines and chemokines, which contribute to the inflammatory response. Microglia are the CNS’s resident immune cells activated in MS. These cells produce cytokines and phagocytosed myelin and can contribute to the inflammatory response by generating ROS and NO.Motor incoordination, muscle weakness, cognitive impairment, depression, optic neuritis, and other MS symptoms are caused by CNS demyelination. 

Abbreviations: Th, T-helper cells; BBB, blood-brain barrier; OGDs, oligodendrocyte cells; APC, antigen-presenting cells (immune response-mediating immune cells).

The diagram illustrates that MS is related to a neurotransmitter imbalance, which results in increased glutamate, an excitotoxic neurotransmitter, and decreased serotonin, acetylcholine, dopamine, and GABA levels. Glutamate elevation and GABA inhibition exacerbate calcium excess, resulting in neuronal excitotoxicity and mitochondrial cell failure. The increasing concentration of ROS and NO causes additional DNA damage and neuronal death, eventually altering myelin sheath development. Infections, vitamin D deficiency, smoking, toxic chemicals and foods, gliotoxins (ethidium bromide, lysolecithin, calcium ionophores, sixaminonicotinamides), genetic 6p21, viruses (EBV, HHV-6), and bacteria (borrelia buradoferi) are all environmental factors that contribute to the onset and progression of MS. While vitamin D deficiency is thought to have an effect on the immune system and increase the risk of developing autoimmune diseases, infections can trigger an immune response and cause inflammation. Smoking also enhances inflammation and oxidative stress, which may exacerbate MS symptoms. To summarize, MS is a complex disorder caused by inherited and environmental elements. These elements can cause immune system failure, oxidative stress, and neurotransmitter abnormalities, all of which contribute to the start and progression of the condition. These essential components cause the release of inflammatory cytokines such as IL-1β, IFN-, IL-23, IL-6, iNOS, MCP-1, and TNFα-2, eventually leading to an attack on neuronal oligodendrocyte cells and the breakdown of the myelin sheath.

Abbreviations: MS, multiple sclerosis; GABA, gamma-aminobutyric acid; ROS, reactive oxygen species; NO, nitric oxide; EBV, Epstein-Barr virus; HHV-6, human herpes virus; IL, interleukins; iNOS, inducible nitric oxide synthase; MCP-1, monocyte chemoattractant protein-1; TNF, tumor necrosis factor.

## 4. Diagnostic Challenges

### 4.1. Misdiagnosis

An early MS diagnosis may significantly improve long-term patient outcomes. Early MS diagnosis and therapy are critical for minimizing disability and maintaining general health in patients [3]. In the last 20 years, there has been a deliberate effort to update and improve diagnostic criteria to be employed more quickly [88]. When people with MS do not get an early diagnosis because of diseases like Schilder’s disease, Eale’s syndrome, sarcoidosis, CNS lupus, etc., it is not a given that they will start treatment when they get to neurosurgeons [89]. Eale’s syndrome, also known as a styloid syndrome, is a rare condition that is typically characterized by sudden, sharp nerve pain in the pharynx, the back of the throat, and the base of the tongue that is brought on by swallowing, moving the jaw, or turning the neck. Eagle syndrome was first described by American otolaryngologist Watt Weems Eagle in 1937. This is caused by a calcified stylohyoid ligament or an extended styloid process, which affects the function of peripheral nerves and results in pain and trouble swallowing [90,91,92].

Despite significant advances in diagnostic procedures for MS over the last few decades, there is currently no viable biomarker [93]. According to specific research, up to 58% of peoplewith optic neuritis will develop MS within 15 years [94]. According to a new Indian study, aquaporin-4 (AQP4) or myelin oligodendrocyte (MOG) antibodies are found in 50% of neuromyelitis optica spectrum disorders (NMOSD) [95]. MS is difficult to diagnose since its subtypes, pseudo-relapses, and clinical presentation vary immensely among patients [96]. Neuromyelitis optica (NMO), a kind of MSinvolving the spinal cord and optic nerve demyelination, is a severe disease affecting people worldwide, including India [3]. The McDonald’s criteria are also used to define PPMS. It must have been present for over a year before it canbe identified. Two criteria must be met simultaneously: two or more T2 hyperintense lesions in the spinal cord, CSF-specific oligoclonal bands, and one or more T2 hyperintense bands in more than one MS-typical location [97]. In one study, better and more general training on how to use MS diagnostic criteria was taught to reduce MS misdiagnosis and raise the larger question of how best to train neurology residents (NR) in training and doctors in practice on the clinical diagnostic criteria for MS [98]. In conclusion, a correct diagnosis is essential to treating and managingthe condition effectively. A thorough assessment by a medical professional with knowledge of MS and continued communication and education will help guarantee the best patient outcome.

### 4.2. MS Misinterpretation

Magnetic resonance imaging (MRI) is vital for diagnosing and treating illnesses such as MS. An MRI of the CNS can help enhance MS treatment. MRI still has a substantial deficit in consistency between the location of lesions and how they appear clinically [99]. Furthermore, regarding sensitivity and specificity for MS diagnosis, the quantity and location of lesions vary significantly depending on MRI. Except for academic institutions, long-term patient follow-up and record-keeping remain impediments to data gathering and analysis. 

In India, the capacity to recognize, diagnose, and monitor MS has been significantly hampered by a lack of knowledge, epidemiological monitoring, and limited diagnostic abilities. MRI is only available in most urban areas with teaching hospitals and for-profit, private institutions that see patients from the highest socioeconomic strata [18,100]. Before the introduction of MRI, there were two clinical subtypes of MS in Asia: the “Western type of MS”, which has extensive lesions, and the “Asian type of MS”, which has lesions confined to the spinal cord and optic nerves (also known as “opticospinal MS”). The MRI abnormalities of Indian MS patients closely resemble those reported in the West. An adequate MRI procedure should be followed for MS diagnosis and follow-up.

Time and effort can be significantly saved by being aware of the MRI lesions that characterize MS and applying the most recent diagnostic criteria in conjunction with the fixed imaging process. Using more recent imaging sequences and stronger magnets inchallenging situations can be a problem-solving tool [101]. Evoked potential studies, particularly visually evoked potentials, are another type of supportive laboratory testing [31]. The presence of OCB in the CSF predicts but does not prove the diagnosis of MS. In the experiments reported by Jena et al. in this matter and several other studies from India, the yield of positive OCB was low [36]. It is critical to clarify the procedure used to identify OCB. The success rate of isoelectric focusing is likely to be much higher.

### 4.3. Biomarker Development Challenges

MS biomarkers should be cost-effective, related to the biology or pathophysiology of the disease, such as inflammatory activity or the degree of neurodegeneration, demyelination, or remyelination, and reliably measured using accurate and robust tests across multiple locations to be successfully implemented in clinical settings [102,103]. Most of the biomarker development process is in the discovery and validation phases [104]. Omic technologies are frequently used in the innovation process to assess a small sample of well-classified patients [105]. To further validate the biomarker’s findings, rigorous statistical methods and careful replication of these results in an independent cohort are required, as the sample size in the discovery phase is typically small. Multiple rounds of validation are required beforetransferring a biomarker from the laboratory to the clinic [106]. 

MS requires lesions in at least two sections of the central nervous system, including the spinal cord, brain, and optic nerves, as well as evidence that the insult occurred at two different times, as confirmed by clinical history or MRI [9,107]. Developing reliable biomarkers for MS remains challenging, but progress has been made in identifying potential markers. Continued research is needed to validate and refine these biomarkers and identify new markers that could improve this complex disease’s diagnosis, monitoring, and treatment.

### 4.4. Treatment Costs

In India, the total cost of MS is unknown. The average yearly cost of disease-modifying drugs in the United States is $60,000, while insurance coverage varies [108]. According to World Bank estimates, India’s average annual per capita income in 2021 will be around $2277. Currently, India has a small insurance market. Approximately 15% of people have insurance, with the remaining 85% paying out of pocket [109]. 

The majority of the population’s insurance coverage is given through government programs and is accessible to government employees, according to the Insurance Regulatory and Development Authority (IRDA). The World Health Organization (WHO) reports that India spent the least amount of its GDP on healthcare in 2015 of any of the BRICS countries (Brazil, Russia, India, China, and South Africa), at 4.0% [110]. Based on the information presented above, we can assume that it will urge the Indian healthcare system to strengthen and enhance the healthcare system to reduce the occurrence of such diseases.

## 5. Currently Available Diagnoses of MS

### 5.1. CSF andSerum

#### 5.1.1. Oligoclonal Bands (IgG) and (IgM)

Patients with oligoclonal bands on immunoglobulin tests can be identified when testing blood serum and cerebrospinal fluid [111]. OCBs are found in the CSF of more than 95% of peoplewith MS. They are not found in their serum, making this a significant diagnostic indicator [112]. The IgG-type (OCGB) oligoclonal bands generated by B cells may be the most significantbiomarker associated with CNS demyelinating disorders. The OCGB is employed as a diagnostic component and is also related to the development of CDMS due to a higher concentration of IgG in the CSF of individuals with CIS. OCGB is required to predict which individuals with Radiologically Isolated Syndrome (RIS) will develop CIS and which will develop MS [113,114]. Although OCGB cannot predict the severity of a second relapse, its presence aids in predicting Optic Neuritis (ON) in MS [112,115]. 

Patients with OCGB in their CSF have higher levels of inflammation response, which causes severe tissue damage, more lesions, and more brain atrophy [116,117]. The more elevated IgG and IgM OCB levels produced intrathecally in MS patients suggest the clonal proliferation of B cells and plasma cells in the central nervous system. The IgG index compares the amount of IgG found in CSF to that seen in serum [112]. OCMB changes inflammatory processes in the brain, resulting in more severe central nervous system (CNS) injuries. It has been related to increased retinal axonal loss and thinning of the retinal nerve fiber layer in MS and its deposition [118,119]. 

Detecting IgG and IgM oligoclonal bands in the CSF is an essential diagnostic criterion forMS. However, OCBs are not specific to MS and can also be found in other neurological conditions. Therefore, the diagnosis of MS should be made based on a combination of clinical symptoms, imaging studies, and laboratory tests, including the presence of OCBs.

#### 5.1.2. NO (Nitric Oxide)

Nitric oxide is a signaling molecule that plays an essential function in pathological disorders such as MS. NO levels in MS patients, CSF, and serum were higher than in non-inflammatory neurological diseases [120]. MS causes inflammatory lesions with higher-than-average NO levels. These higher levels are caused by the inducible form of nitric oxide synthase (iNOS) found in cells such as macrophages and astrocytes. MS patients have higher nitrate and nitrite levels in their CSF, blood, and urine, indicating that NO is being produced. Circumstantial evidence implies that NO is implicated in the collapse of the blood-brain barrier, oligodendrocyte damage and demyelination, axon degeneration, and other signs of illness. It may also contribute to function loss by affecting axonal conduction.

Regardless of these factors, NO generation in MS does not always have a negative impact because it has several immunomodulatory effects [121,122]. Even though peroxynitrite, not NO, is more likely to cause NO neurotoxicity, NO plays a vital role in the pathogenic process of demyelinating disorders. NO can still impact oligodendrocyte energy metabolism by damaging mitochondrial DNA, membranes, and respiratory chain complexes. During MS, NO is primarily responsible for demyelination, axonal degeneration, and cell death [123]. By knowing all of this important information, we can conclude that it is possible that targeting NO could be a potential therapeutic strategy for treating MS, but this remains to be determined through further investigation.

#### 5.1.3. Serum Glial Fibrillary Acidic Protein (sGFAP) 

Astrocytes mainly express GFAP, an intermediate filament (IF) protein also seen in ependymal cells [124]. Increased GFAP levels have been associated with astrocyte death, astrogliosis, and severe disability in MS patients, according to research [78]. GFAP has been detected in enteric glial cells, part of the enteric nervous system, and non-myelinating Schwann cells in the PNS (ENS) [125]. In one investigation, serum GFAB levels were significantly higher in PPMS patients than in RRMS patients [126]. s-GFAP remained considerably greater in PPMS after age and disease duration were incorporated into the multivariate analysis. After controlling for disease duration, a recent relapse’s prevalence was related to lower s-GFAP levels in the RRMS group. This link, however, was not statistically significant [126]. A study discovered that GFAB might be a biomarker for the severity of the MS illness, with considerable amounts identified in patients with severe neurological disabilities [127]. Prior research indicates that a high blood GFAB level may be an excellent therapeutic option for avoiding MS development.

### 5.2. Saliva and Serum 

#### MBP (Myelin Basic Protein)

MBP is the second most prevalent protein in the myelin sheath of the central nervous system. Due to its crucial role in the production and completion of CNS myelin, MBP has been identified as a functional myelin molecule, and it is required for proper vertebrate function [128]. Saliva is an ideal analytical fluid because it is easy to collect and maintain and is less expensive than other fluids used in medical labs [129]. 

MBP concentrations in the blood and saliva were lower in MS patients with a mean age of 35.7 ± 7.9, making them potential indicators for the condition. Researchers also discovered that MBP levels in the serum and stimulated saliva of women with MS who used the Elisa test were much lower than in healthy women. Furthermore, there is a strong correlation between the amount of MBP in the saliva stimulated and the amount in the blood [129,130]. Further research is needed to determine its potential as a reliable diagnostic tool or marker of disease activity.

### 5.3. Urine Metabolites 

Many biomarkers have been discovered in urine, which has the potential to be a quick, inexpensive, non-invasive, and effective diagnostic method for a wide range of human diseases. As a result, urine metabolites may provide a potentially beneficial tool for diagnosing MS and evaluating the in vivo efficacy of MS therapy candidates [131]. Sinceurea is essential for maintaining ammonia and amine nitrogen balance through its function in amino-acid metabolism, decreased urea-cycle activity can result in hyperammonemia, a key contributor to some types of acute neurological disorders [132,133]. Malnutrition reduces protein ingestion, causing urea levels to drop considerably. MS patients exhibited higher malnutrition rates than those with other chronic conditions [131]. 

Uric acid, present in extracellular fluid as sodium urate, is thought to be responsible for more than half of plasma’s antioxidant action. Research suggests that uric acid may have neuroprotective effects by scavenging reactive oxygen and nitrogen radicals like peroxynitrite. In both in vivo and in vitro investigations, uric acid has demonstrated a substantial antioxidant impact on neurons [134]. Urine neopterin, nitric oxide metabolites, and urinary melatonin levels, which were lower in MS patients, have all been identified as relevant urinary indicators for diagnosing MS [135,136]. Hippuric acid, a conjugation of benzoic acid and glycine, has long been a significant human metabolite.

On the other hand, Hippurate has various uses; it is a biomarker for high-dose exposure to toxic chemicals like toluene and is frequently employed as a measure of renal clearance [131,137]. The gut flora is an essential factor influencing the hippurate synthesis rate. The study found numerous links between IBD (Crohn’s disease and ulcerative colitis) and MS, which affects gut flora [138,139]. The quantity of hippuric acid in the urine of MS patients was found to be much lower. This reduced hippuric acid level could be due to an innate fault in glycine conjugation or a lack of benzoic acid [131]. Using a colorimetric technique, a clinical study found that MS patients had significantly reduced urine metabolite values for urea, uric acid, and hippuric acid. These metabolites also decreased the SPMS pattern significantly compared to the RRMS pattern [131]. Based on these significant findings, we can conclude that urine metabolites may be an excellent therapeutic target, but further investigations must be confirmed.

### 5.4. Tear

#### 5.4.1. Alpha-1 Antichymotrypsin

Researchers determined in a study that tear fluid could be regarded as a significant biological resource for detecting MS biomarkers [140]. Tear fluid appears to be a fluid of interest in discovering novel biomarkers for therapeutic purposes. For patients, the collection technique is easy, painless, and non-invasive. Furthermore, the composition of tears provides information about the CNS and other systemic approaches, as well as the state of the ocular and underlying tissues [141]. Salvisberg et al. conducted three quantitative experiments comparing MS patients, tears to healthy controls. Out of 42 differential proteins, he discovered that alpha-1 antichymotrypsin, an acute inflammatory protein, was the only one that showed a significant increase across all tests [141]. 

High alpha-1 antichymotrypsin levels in tears replace standard lumbar punctures as a possible MS diagnostic [142]. Based on the previous vital findings, we can conclude that alpha-1 antichymotrypsin in tear fluids may be an excellent therapeutic target for treating MS.

#### 5.4.2. OCB

Tear fluid and CSF have been proposed to detect OCB [143,144]. The existence of OCBs in tears has been explored in MS patients, and various studies have found OCBs in tear samples. According to one study, 55% of MS patients had OCB in their tears, whereas another study showed 72% [145]. 

The clinical significance of OCBs in tear samples is unknown, as is whether their presence corresponds with disease activity or severity. Some research has shown that OCBs in tears may reflect the underlying inflammatory process in MS, whereas others have found no link between OCBs in tears and disease activity [146,147]. While the presence of OCBs in tear samples may be a useful diagnostic marker for MS, more research is needed to determine its clinical significance and potential use as a biomarker for disease activity and progression.

### 5.5. Predictive Biomarkers

#### 5.5.1. HHV-6 

By establishing large amounts of human herpesvirus-6 (HHV-6) viral expression in oligodendrocytes proximal to MS plaques, the experimental findings suggest a direct causative connection between HHV-6 infection and the development of MS [112]. The presence of viral DNA in the brain and CSF of MS patients adds to the evidence of HHV-6 neurotropism in the disease. Clinical case-control investigations also discovered that oligodendrocytes from MS patients had higher levels of viral mRNA and proteins and higher levels of HHV-6 gene expression [112,147]. Although the evidence for HHV-6 infection is not as strong as that for EBV, it may be more common in MS patients. However, some studies have found that HHV-6 IgG titers in MS patients were higher than in matched controls at the time of disease onset. Other research has found no significant differences [148,149]. The previous study found that higher HHV-6 IgG levels were associated with a higher risk of relapse in established MS patients, implying that this link may extend to MS conversion and relapse [130,148]. Based on these significant findings, we can say that HHV-6 is significantly associated with MS disease.

#### 5.5.2. Antibodies against MOG and MBP

Autoantibodies can damage the myelin sheath if they damage oligodendrocytes, which are necessary to produce myelin in the central nervous system. Myelin protein-specific antibodies in the serum indicate an autoimmune attack on CNS myelin. As a result, anti-myelin autoantibodies such as MOG and MBP found in the serum of CIS patients may be used as diagnostic markers [112]. Anti-MBP antibodies as a kid have also been linked to an increased risk of demyelinating encephalomyelitis [150]. Anti-MBP antibodies were also discovered in the blood of peoplewith CIS who later developed MS [151].

However, Kuhle et al. discovered no apparent link between the presence of anti-MOG and anti-MBP and the progression of CIS to MS, rendering the studies inconclusive [130,152]. These previous findings suggest that myelin antibodies like MOG and MBP are associated with the progression of the neurodegenerative disease MS.

### 5.6. PrognosticBiomarkers 

#### 5.6.1. Chitinase-3-Like-1 (CHI3L1)

CHI3L1 and CHI3L2 proteins bind chitin and are physically similar to chitinases but cannot hydrolyze chitin. In MS brain tissue, CHI3L1 (also known as YKL-40) and CHI3L2 are expressed in astrocytes in white matter plaques and normal-appearing white matter, respectively, with CHI3L1 being detected in microglia [153,154,155]. CHI3L1 has garnered the most attention about MS among the three members of this family. The function of CHI3L1 in tissue remodeling and chronic inflammation is unknown [155,156]. Through unbiased proteomic screening and ELISA testing of CSF, elevated levels of CHI3L1 have been linked to optic neuritis, CIS, and MS [153,157,158,159]. 

Higher levels of CHI3L1 in CSF (cutoffs of 100, 170, or 189 ng/mL, depending on the study) are associated with a greater likelihood of transition from CIS or optic neuritis to clinically definite MS [153,160,161]. Based on these significant findings, we can say that using CHI3L1 as a biomarker for MS holds promise, but more research is needed to fully understand its clinical significance.

#### 5.6.2. Neurofilaments 

Serum neurofilament light chain (NFL) is a neuronal damage biomarker used to forecast the course of the disease in MS patients and track disease activity and drug response. Individuals cannot utilize sNfL as a diagnostic tool since no representative reference values account for how sNfL varies as people age [162].

Neurofilaments are heteropolymers made up of low-molecular-weight (neurofilament light [NF-L]), medium-molecular-weight (neurofilament medium [NF-M]), and high-molecular-weight protein subunits (neurofilament heavy [NF-H]). They are intermediate filaments found only in neurons. They are attractive candidates for biomarkers because of their stability and high concentration in central nervous system tissue. Neurofilaments are released into the extracellular space when an axon is damaged. These levels of neurofilament in the blood or cerebrospinal fluid (CSF) are assumed to reflect the amount of axonal injury. In CSF, NF-L and phosphorylated NF-H (PNF-H) levels are elevated in MS, especially during relapses [152,163]. Protein stability and assay sensitivity could explain the disparity in NF-L and NF-H levels [164]. NF-L is assumed to reflect early, acute, inflammatory-mediated axonal damage because of its relationship with inflammatory disease and its less accurate correlation with the course of impairment [165]. Based on this critical information, we can conclude that serum NFL could be a therapeutic target for detecting and treating MS. These findings suggest that CSF NfL levels may be a valuable biomarker for monitoring disease activity and progression in MS.

#### 5.6.3. MicroRNAs (miRNAs)

Mi-RNAs are noncoding RNA molecules that regulate gene expression by blocking or stimulating post-transcriptional protein synthesis [166,167]. Mi-RNA dysregulation has been proposed as a possible marker of disease development in MS. It may significantly affect how the disease functions [168,169]. Most biofluids have been found to contain miRNAs identified using various techniques, including quantitative PCR, miRNA array analysis, short non-coding RNA cloning, and next-generation sequencing [170,171]. Based on the previous findings, we can conclude that miRNAs have been shown to play a significant role in the pathogenesis of MS.

#### 5.6.4. CXCL13

CXCL13 is a small chemokine that is essential for the formation of lymphoid follicles in secondary lymphoid organs. It was identified as a B-lymphocyte chemoattractant for the first time in 1998 (SLOs) [26]. CXCL13 chemokine levels are higher in MS models with an inflammatory CNS [172,173]. CXCL13 intrathecal production in MS animals and biopsied or autopsied MS brains can be directly measured by measuring CXCL13 mRNA or detecting CXCL13 by immunostaining [174]. Sincetissue is unavailable in people with MS, most studies have looked at CXCL13 levels in bodily fluids like serum and CSF. Most studies have employed enzyme-linked immunoassays (ELISAs) to quantify CXCL13. By measuring CXCL13 in the CSF, this test has been used to diagnose Lyme neuroborreliosis (LNB) [175,176,177,178], an infection of the CNS with the spirochete Borrelia burgdorferi [179]. SinceMS inflammation is localized to the central nervous system, most of these studies have focused on CSF. Despite its sensitivity issues, ELISA has been the most commonly used method [159,180,181]. Based on these significant findings, it is clear that CXCL13 is involved in the pathogenesis of the disease and may have clinical utility as a biomarker. Further research is needed to fully elucidate how CXCL13 contributes to MS and determine whether CXCL13-targeted therapies may effectively treat the disease.

#### 5.6.5. CXCL12

It controls hemopoiesis and is a strong chemoattractant for several immune cells, including monocytes, T cells, B cells, and plasma cells. In addition, CXCL12 plays complex roles in neurobiology. It is essential for the development of neural guidance. In a study, it was discovered that metalloproteases, which are prevalent in multiple sclerosis lesions, can cleave CXCL12, making it a neurotoxic mediator of axonal damage [174]. B cells move to the brain under the control of the particular chemokine CXCL12, where they develop into plasma cells that produce antibodies. B cells are capable of acting as cells that deliver antigens.

Additionally, they can release cytokines that promote inflammation. Anti-CD20 monoclonal antibodies effectively decrease the activity of inflammatory diseases like MS by depleting CD20+ B and CD20+ T cells. Finally, the overexpression of CXCL12 causes the destruction (depletion) of anti-CD20 antibodies, leading to neuroinflammation. In MS, CXCL12 levels are higher in actively demyelinating lesions, facilitating B cell entry into the CNS [182].

#### 5.6.6. SIRT-1

**Figure 4:**
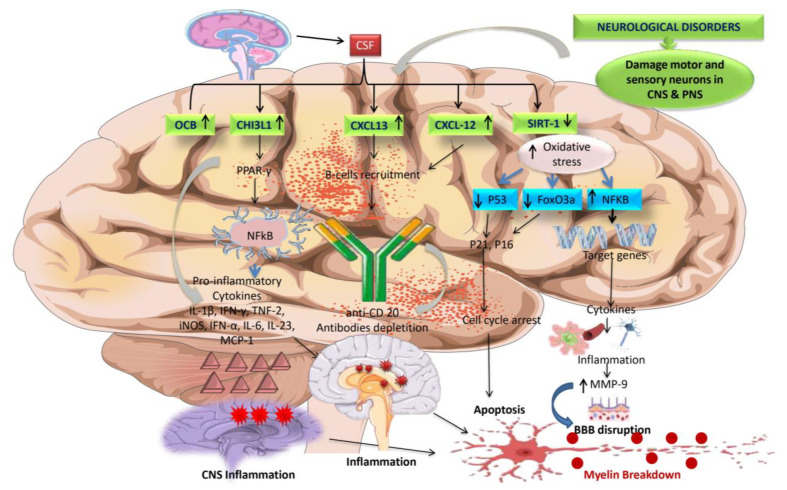
Alteration in the expression of major cellular and molecular indicators in MS CSF.

A vital protein for cellular life known as SIRT1, a class III protein deacetylase, also prevents oxidative stress. SIRT1 can activate the FoxO pathways to promote the production of antioxidants. Additionally, SIRT1 inhibits NF-KB signaling, a primary inducer of inflammatory responses, such as via the inflammasome pathway, in contrast to ROS. Reduced SIRT1 activity boosts NF-B signaling, encouraging inflammatory responses [183] (Figure 4). Other molecular changes, such as gene expression modification, which affects neural plasticity and inhibits Th17 cells, and interleukin-1, which can exacerbate brain illnesses, include SIRT-1 dysregulation, which targets transcription factors. According to preclinical and clinical research, the overexpression of SIRT-1 decreases autoimmunity, neurodegeneration, and neuroexcitation [27]. In MS disease, SIRT-1 levels decreased, leading to neuroinflammation and further demyelination.

#### 5.6.7. PI3K/AKT/mTOR 

The phosphatidylinositol-3-kinase (PI3K)/AKT/mTOR signaling system regulates proper cell growth, metabolism, and survival [184]. Through intracellular signaling, this pathway governs cell activation, proliferation, metabolism, and apoptosis. Evidence suggests that changes in the PI3K/AKT/mTOR pathway may increase vulnerability to autoimmunity [184,185]. Activation of this pathway in immune cells can promote their survival, migration, and production of pro-inflammatory cytokines, thereby contributing to the autoimmune response observed in MS [186].

#### 5.6.8. EBNA igG

A study discovered a link between Epstein-Barr virus nuclear antigen 1 (EBNA-1) IgG and gadolinium-enhancing lesions, implying a relationship between EBV infection and MS disease activity. Higher levels of EBNA-1 IgG in CIS patients who convert to CDMS within 5 years may be a valuable biomarker in the future, but more research is needed. In the current investigation, higher EBNA-1 IgG titers were shown to be linked with the development of Gd+ lesions on MRI in arecent study [187].

#### 5.6.9. JAK/STAT and PPAR-γ

**Figure 5:**
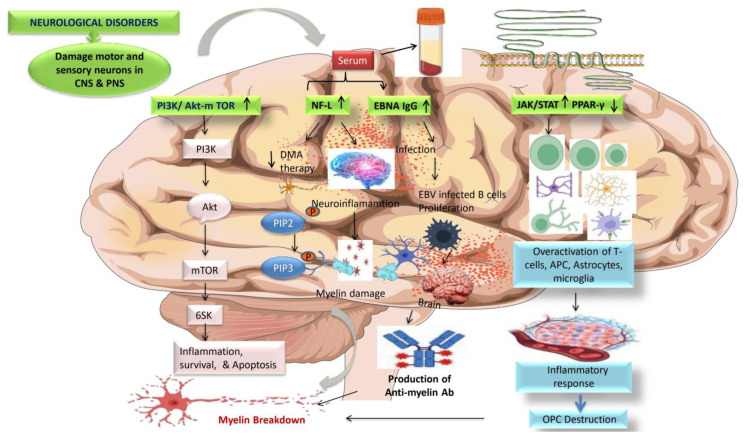
Changes in the expression of significant cellular and molecular indicators in MS patients, CSF and blood serum.

The JAK/STAT signaling system is a signaling cascade that regulates immunological responses, cell development, and differentiation. This route has been found to be dysregulated in a variety of autoimmune disorders, including MS. JAK/STAT signaling activation has been linked to increased production of pro-inflammatory cytokines such as interferon-gamma (IFN-γ) and interleukin-6 (IL-6), both of which contribute to the inflammatory response in MS [188]. PPAR-γ expression and activity in MS have also been studied. Further, its expression is lower in the immune cells of MS patients compared to healthy individuals in studies. This decreased expression may lead to immune response dysregulation and the maintenance of inflammation in MS [189,190]. In conclusion, both the JAK/STAT pathway and PPAR-γ have a role in the etiology of MS. JAK/STAT pathway dysregulation leads to aninflammatory response. In contrast, decreased PPAR-γ expression may lead to the persistence of inflammation in MS (Figure 5). 

Table 1 contains essential cellular and molecular biomarkers that are potential therapeutic targets for treating and preventing multiple sclerosis. CSF, plasma, and blood serum samples from MS patients were discovered to be modulated. 

**Table 1:** Current diagnostic biomarkers available for MS prophylaxis.

S.N.	Biomarkers	Biological Samples	Ranges (Units)		Types/ Category of MS Patients	Patients	References
			MS	Normal ranges		Age and Gender	
1.	Chitinase-3-like-1(CHI3L1)	CSF	22.7 pg/mL	13.4–57.9 pg/mL	RRMS	Mean Age: 30.3 ± 9.25 years Gender: 46Fand13M	[191]
		Serum	20.2 pg/mL	9.8–75.9 pg/mL	CIS		
2.	CXCL13	CSF	35 mg/dL	<30 mg/dL	RRMS	MeanAge:34 ± 8.3 years Gender: 5F and3M	[191]
3.	Neurofilament	Plasma	11.4 pg/mL	7.5 pg/mL	RRMS	Mean age: 40 Gender: 328F and 344M	[192]
4.	EBNA1 IgG	Serum	310.91 U/mL	177.81 U/mL	RRMS	Mean age: 29.69 Gender: 27M and 48F	[193]
5.	OCBs	CSF	5–7 bands	1 band	RRMS, SPMS	Mean Age: 35 Gender: 14F and 8M	[194,195]
6.	miRNA	Plasma	±>1.5 fold change	−5.30 to +1.94 Fold range	RRMS	Mean Age: 31.5 Gender: 20F and 16M	[196]
7.	Alpha-1 antichymotrypsin	Tears	1.6 ng/L	2.5 ng/L	RRMS = 25, PPMS = 4, SPMS = 1	Mean age: 42.4 ± 15 Gender: Males	[141]
8.	Myelinbasic protein (MBP)	Serum	1055 ng/L	2750 ng/L	RRMS	Mean age: 36.8 ± 4.2 Gender: Females	[129]
		Saliva	475 ng/L	575 ng/L	RRMS		

The expression of the CSF Chitinase-3-like protein (CHI3L1) biomarker is increased in MS patients. Astrocytes and microglia have been discovered to manufacture them. Increased biomarker expression in CSF regulates PPAR-γ, resulting in the production of pro-inflammatory cytokines. These cytokines cause chronic inflammation in the CNS. Other biomarkers, CXCL-13 and CXCL-12, have elevated CSF expression, attracting B-cells into the CNS, depleting anti-CD20, and causing chronic inflammation. The decreased SIRT-1 expression in CSF resulted in the degradation of the OGDs. The pathway involves higher oxidative stress levels due to lower levels of tumor suppressor p53 and FoxO3a, a novel regulator of non-oxidative glucose metabolism, acting via transcription of p21 and p16, both essential regulators of G1/S cell-cycle checkpoints. They then block the CDK4/6-cyclin D and CDK2-cyclin E complexes, modifying cell death further. Increased inducible transcription factor NF-KB signaling production is also an essential modulator of the inflammatory response. It acts on target genes, producing cytokines and chemokines that cause inflammation, which raises the level of MMP-9, which has been linked to BBB damage and, ultimately, myelin sheath collapse. 

Abbreviations: MS, multiple sclerosis; CSF, cerebrospinal fluid; PPAR-γ, peroxisome proliferator-activated receptor; anti-CD20, monoclonal Ab; SIRT-1, sirtuin1; OGDs, oligodendrocytes; CDK, cyclin-dependent kinase; NF-KB, nuclear factor kappa B; MMP-9, matrix metalloproteinase 9; EBV, Epstein-Barr virus.

The figure illustrates that increased expression of the PI3k/Akt-mTOR pathway is responsible for inflammation, cell death, and proliferation, all of which are associated with MS development. Increased production of blood biomarkers such as NF-L and EBNAIgG causes neuroinflammation and EBV infections in the CNS, respectively, activating anti-myelin antibodies—the ensuing NF-L and EBNAIgG induce myelin to destruct. Motor neuron illnesses like MS have direct physiological and pathological effects due to overexpression of the JAK/STAT signaling system. JAK/STAT signaling is used by cytokines such as IL-17, IL-6, IL-12, TNFα, and IFN-γ to activate self-reactive CD4+ T cells and differentiate them into Th1 phenotypes that overactivate immune responses in the brain. PPAR-γ is essential for modulating the immune response because it has an anti-inflammatory effect by inhibiting macrophage and cytokine activation. Additionally, it governs the T-cell’s intrinsic molecular mechanism, which specifically controls Th17 differentiation. Increased expression of PPAR-γ is linked with neuroprotective action by reducing JAK/STAT-mediated overactivation of glial cells, decreasing interleukins, and preventing the formation of Th1 cells.The figure illustrates that PPAR-γ and JAK/STAT signaling dysregulation leads to the inflammatory response destroying the OPC cells, further leading to myelin damage.

Abbreviations: NF-L, neurofilament light chain; EBNAIgG, Epstein-Barr virus nuclear antigen; CSF, cerebrospinal fluid; PPAR-γ, peroxisome proliferator-activated receptor; DMA, disease-modifying agent; PI3K, phosphoinositide 3-kinase pathway; Th17, T-helper cell 17; JAK/STAT, Janus kinase signal transducers and activators of transcription.

## 6. Treatment Challenges

### 6.1. Inadequate Treatment Initiation

Early treatment can reduce the cost of care for people with MS while improving their health anddecreasing disability development [197]. Patients are frequently ignorant of the expenses connected to MS and the consequences of prolonged therapy. Direct and indirect costs rise as the disease advances and the degree of disability increases. This cost rise is typically tied to relapses and productivity expenses rather than the direct cost of using DMTs [198,199]. When viewed from an individual standpoint, the cost issue is complicated. Patients typically discontinue therapy when their cost-sharing threshold is exceeded [89]. Treatment approaches that make it difficult for patients to begin taking their medications as recommended are more likely to trigger a relapse, accelerate disease development, and result in lifelong disability [200]. Early MS treatment can slow the progression of disability and enhance long-term clinical outcomes [201,202]. It also improves the ability to work and is associated with better socioeconomic outcomes [203]. As a result, early therapy for MS may reduce the overall cost of illness (COI) [204].

In conclusion, the inadequate treatment initiation of MS in India is a complex issue that may require a multi-faceted approach. Improving awareness about the disease among patients and healthcare providers, reducing the cost of MS medications, and increasing access to specialized healthcare services and professionals are crucial steps in addressing this issue.

### 6.2. Lack of Continuous Treatment

In India, there is a lack of continuing treatment.Untreated MS can exacerbate or intensify the disease’s devastation forindividuals. According to estimates, 90% of patients not treated 20–25 years after becoming ill would eventually become incapacitated [205,206,207]. Treatment for illnesses such as MS must be ongoing to be effective. Poor treatment adherence can also be caused by observable ineffectiveness, budgetary constraints, patient suffering, and a patient’s desire to try available alternative treatments [208,209,210]. The most significant cause of noncompliance among MS patients is the delayed onset of symptoms following the initial diagnosis. Following an initial MS diagnosis, some patients do not experience a significant relapse or the emergence of new symptoms for several months or years, increasing the likelihood that the patient will refuse to accept the disease, comprehend the importance of routine therapy, and adapt to it [209,210].

Non-adherence or poor adherence to treatment regimens is the most common issue in treating individuals with chronic diseases. Non-adherence, side effects (such as fatigue, flu-like symptoms, and reactions at the injection site), a patient’s lack of awareness or neglect, or problems with injections (such as fear, anxiety, pain, and discomfort) can all result from complicated treatment plans [208,210]. In the case of MS, adherence to DMDs ranges from 41% to 93% [209]. Over two years, patients who received DMT regularly had a much lower rate of severe relapses and overall treatment costs [211]. MS patients who get DMT treatment for the first year stick to their regimen more regularly than those with other chronic diseases such as epilepsy, rheumatoid arthritis, or Parkinson’s disease [212].

Patients who adhere effectively to disease-modifying drugs (DMDs) have a lower risk of recurrence, fewer hospitalizations, and a better quality of life [213]. Based on these significant findings, we can say that the lack of continuous treatment for MS in India is a significant health and socioeconomic issue that demands the attention of policymakers, healthcare practitioners, and the general population. Raising awareness and expanding access to treatment may be helpful for MS patients in the continuous treatment of the disease in India.

### 6.3. Patients with Concomitant Liver Illness or a History of Drug-Induced Liver Damage

The most common reason that drug development is halted or indications are restricted after a medicine is approved for sale is drug-induced liver injury (DILI). It has received a lot of interest from regulators, industries, researchers, and clinicians [214]. For example, in 2018, the European Medicines Agency (EMA) recalled the monoclonal antibody daclizumab from the market due to serious and potentially fatal immunological reactions (liver injury and encephalitis) [215].

Toxic medicines, fatty infiltration, viral infections, and autoimmune illnesses can all cause liver damage in people with MS [216]. The possibility of liver impairment may limit the patient’s therapy options. Some DMTs, such as alemtuzumab, fingolimod, interferons, mitoxantrone, and teriflunomide, are associated with a risk of liver damage [217]. DMT treatment can potentially reactivate chronic liver disorders and autoimmune hepatitis [217,218]. In a Canadian retrospective analysis, about 2% of MS patients who received interferon beta had drug-induced liver damage [219]. Based on this information, we can say that drug-induced liver damage or a history of liver illness is an emerging challenge for the new drug development process for treating various illnesses, including MS.

### 6.4. Elderly Patients

While most new MS cases are diagnosed in young adults, the disease’s prevalence rises in middle age. According to current estimates, the peak occurrence of People with MS occurs between 50 and 60, implying that many People with MS are older than the patient populations in critical DMT studies [220,221]. Due to the population aging, faster diagnosis, more access to DMTs, and enhanced supportive care, the prevalence of MS in older persons is growing [222,223,224].

Furthermore, approximately 5% of people with MS develop it after age 50 or later. This type of MS frequently produces motor issues and has a poor prognosis [225,226,227]. Choosing an appropriate drug is already difficult for people with MS. The increasing frequency of co-morbidities, polypharmacy, and immunological senescence in older people adds to the complications. There is evidence that the efficacy of DMT decreases with age in people with MS [228,229]. As people age, the benefit-risk ratio of a DMT may vary, favoring a less effective DMT with a reduced risk of unwanted effects.

Meanwhile, some side effects of highly effective DMTs are more common in older people [230]. Only 12% of 377 people with MS who had been taking DMT for at least five years and were younger than 45 said they would consider discontinuing DMTs if there was no evidence of disease activity [231]. Based on all these vital findings, we can conclude that older adults pose a challenge for healthcare professionals regarding the treatment regimen for neurocomplications and neurodegenerative diseases.

### 6.5. Pregnancy and Family Planning

Pregnancy may provide natural protection when DMT is suspended and is linked to decreased MS disease activity [232]. Except for interferon and glatiramer acetate (GA), all medications should be stopped before attempting to conceive. Receiving DMT increases the likelihood of conception in women. Depending on the DMT, discontinuation could increase disease activity [233,234].

However, most of the safety data on exposure is based on the first month after conception. They are primarily concerned with teratogenic rather than late-term issues, such as immunologic effects [233,235]. MS patient registries show injectable DMTs (GA and IFN-γ) are safe before conception and during the first trimester. However, data on their continuation during pregnancy is limited [236,237,238]. It is recommended to avoid exposure during pregnancy as a precaution unless the advantages to the mother outweigh the hazards to the unborn child. As a bridge therapy when attempting to conceive, it may be necessary to use a less hazardous medicine [233].

In conclusion, women with MS considering pregnancy or family planning must work closely with their healthcare providers to manage their disease and plan for a healthy pregnancy. It is essential to balance the potential risks and benefits of MS treatment with the desire to have children and to make informed decisions about contraception and family planning. With careful management and planning, women with MS can successfully navigate the challenges of pregnancy and family planning.

Table 2 lists clinical and preclinical medications for treating different types of multiple sclerosis, including RRMS, SPMS, and PPMS.

**Table 2:** MS treatments currently available.

S.NO	Nameof Drugs	Dose and Route	Adverse Effect	Patients Type	Duration of Treatment	References
1.	Fingolimod (Peptide)	0.5 mg p.o. daily	Infections, bradycardia, MS relapse, and basal-cell carcinoma.	RRMS	6–12 months	[239,240]
2.	Interferon β-1a (Glycoprotein)	30 mcg (IM), Once a day 22 mcg (SC), TDI	Flu-like symptoms (fever, chills, sweating, muscleaches, and tiredness), skin reaction, depression, anxiety, and liver problems.	RRMS	24 months	[100,241,242]
3.	Interferon β-1a (Glycoprotein)	22 mg, three injections weekly (SC)	Fatigue, allergic reactions, flu-like symptoms, emotional instability, trouble breathing, joint problem, eye problems, and hair loss.	RRMS	6–24 months	[241,243,244]
4.	Interferon β-1b (Non-glycosylated protein)	0.25 mg (SC) q.o.d., 6 weeks	Leucopenia, flu-like symptoms, elevated hepatic transaminases, injection site reactions, headache, fever, malaise, and myalgia.	RRMS	24 months	[242,245,246]
5.	Alemtuzumab (Monoclonal antibody)	12 mg (IV) daily	Infusion-associated reactions(IARs) include headache, rash, nausea, fever, respiratory tract infection, and thyroid disease.	RRMS	12 months	[247,248]
6.	Dimethyl Fumarate (Peptide)	240 mg/kg (p.o.) Twice a day	Abdominal pain, alopecia, back pain, cough, diarrhea, flushing, headache, influenza, paresthesia, and nausea.	RRMS	24 weeks	[249]
7.	Glatiramer acetate (peptide)	20 mg/kg (SC) daily	Post-injection reaction, chest pain, lipoatrophy, and skin necrosis potentially affect the immune response.	RRMS	24 months	[250,251].
8.	Dalfampridine (Pyrimidine analogue)	10 mg/kg twice a day	Asthenia, insomnia, paresthesia, UTI, dizziness, nausea, peripheral edema, back pain, and nasopharyngitis.	RRMS	4–24 weeks	[252,253]
9.	Natalizumab (Monoclonal antibody)	300 mg/kg (i.v.)	Occurrence of PML, fatal cases of neutralizing antibodies, and PML HSV1/VZV reactivation.	RRMS	≥12 months	[202,250].
10.	Ocrelizumab (Monoclonal antibody)	300 mg/kg (i.v.)	HSV1/VZV reactivation, HBV hypogammaglobulinemia, and breastcancer PML (carry over).	RRMS	6 months	[250,254]
11.	Teriflunomide (Enamide)	14 mg/kg (p.o.)	Hepatic events, lymphopenia, neutropenia, thrombocytopenia, hypertension, pancreatic disorders, hair thinning, and GIT events.	RRMS	12 weeks	[255,256]
12.	Siponimod (Alkoxyimino)	0.25–2 mg/kg (p.o.)	Bradycardia, rapid receptor desensitization, decreased absolute lymphocyte count (ALC), lymphopenia, upper respiratory tract infections, pharyngitis, insomnia, and increased alanine aminotransferase.	RRMS	>12 months	[257,258]
13.	Rituximab (Chimeric murine/human monoclonal antibody)	500–1000 mg (IV)	Infusion-related adverse events include rash, fatigue, chills, nausea, and general pain.	RRMS	72 weeks	[259,260]
14.	Mitoxantrone (dihydroxyanthraquinone)	12 mg/kgbody weight every three months	Mild infections, leucopenia, irreversible amenorrhea, congestive heart failure, alopecia, and asymptomatic systolic dysfunction.	RRMS and SPMS	2–3 years	[261]
15.	Azathioprine (Purine analogue)	3 mg/kg daily (p.o.)	GIT disturbance, hepatic toxicity, bone marrow suppression, hepatic toxicity, and increased risk of cancer in MS patients.	RRMS	6 months	[262]
16.	Methylprednisolone (Corticosteroids)	500–1000 mg/daily Oral/i.v.	It may cause interaction with warfarin, reduce the effects of enzyme inducers like anti-epileptic agents, dyspepsia, constipation, euphoria, and altered glucose metabolism.	RRMS	3–5 days	[263]
17.	Cladribine (Purine antimetabolite)	3.5 mg/kg (p.o.) two times, 4 or 5 days of treatment each year	Mild renal impairment, hepatic impairment, contraindicated in patients with moderate or severe renal impairment (creatinine clearance < 60 mL/min), and lymphopenia.	RRMS	2 years	[264,265]
18.	Simvastatin (Statin)	80 mg/kg, per day (p.o.)	Muscle pain, dizziness, fainting, headache, nausea, and digestive problems.	SPMS	24 months	[266,267]
19.	Memantine (Amine)	20 mg/day	Headache, dizziness, agitation, hallucinations, confusion, and diarrhea.	RRMS	52 weeks	[268,269]
20.	Donepezil (Peptide)	10 mg/daily (p.o.)	Nausea, diarrhea, headaches, gastroesophageal reflexes, and loss of appetite.	RRMS	24 weeks	[270,271]
21.	Baclofen (Peptide)	10–100 mcg intrathecal	Dizziness, drowsiness, headache, weakness, and nausea.	SPMS and PPMS	4.9 years	[272]
22.	Ublituximab (Monoclonal antibody)	150–600 mg/kg i.v. infusion	Infusion-related reactions, nausea, upper respiratory tract infection, arthralgia, hypoesthesia, dizziness, fatigue, and diarrhea.	RRMS and SPMS	48 weeks	[273]
23.	Ponesimod (Peptide)	10,20, 40 mg/kg Daily (p.o.)	Increase alanine aminotransferase, nasopharyngitis, headache, upper respiratory tract infections, and alopecia.	RRMS and SPMS	24 weeks	[274,275]
24.	Ofatumumab (Monoclonal antibody)	20 mg/kg (S.C.)	Headache, nasopharyngitis, urinary tract infections (UTI), upper respiratory infections, and injection-site reactions (pain, itching, erythema, and swelling).	RRMS and SPMS	12 weeks	[276]
25.	Monomethyl Fumarate (Non-peptide)	95–190 mg/kg (b.i.d.), orally Delayed release capsule	Flushing and GI adverse events (abdominal pain, diarrhea, nausea, and vomiting).	RRMS and SPMS	5 weeks	[277]
26.	Laquinimod (Amide)	0.3–0.6 mg/kg (p.o.)	Elevation of liver enzymes, back pain, abdominal pain, cough, dizziness, headache, diarrhea, and respiratory pain.	RRMS and PPMS	12–24 months	[278,279]

## 7. Future Perspectives

### 7.1. Early Diagnosis of MS

Early detection is critical for slowing disease progression and disability. When diagnosing MS patients, the advisory board suggested considering the following essential factors:

*Follow-up with the MS patient*

All patients must be evaluated clinically using the Expanded Disability Status Scale (EDSS). Patients may contact the doctor for causes other than MS if they are experiencing health issues. In the later stages of the disease, one MRI peryear may be optimal. According to specialists, real-world experience, MRI lesions are not always connected with MS progression (for example, when the condition progresses from relapsing-remitting MS to SPMS [280].

*Referrals to neuroscientists from other doctors*

This should be performedas quickly as feasible for individuals with optic neuritis. Furthermore, patients with paroxysmal symptoms are more likely to develop MS later in life [281,282]. As a result, paroxysmal symptoms should not be overlooked.

*Modern Diagnostic Techniques Adoption*

Modern diagnostic procedures such as magnetic resonance imaging (MRI), cerebrospinal fluid analysis, and optical coherence tomography, which can aid in the early identification of MS, must be widely adopted. Sincethe cost of these diagnostic tests can be prohibitive for some patients, it is critical to work on making them more accessible and affordable.

*Exclusion of illnesses with comparable clinical manifestations*

An MRI should be used to rule out any other causes of lesions that resemble MS before a patient is diagnosed. MS and NMO should not be mistaken because they have similar clinical signs and symptoms, such as fatigue, sadness, and dizziness. MS should be diagnosed using particular diagnostic standards from the new NMO diagnostic criteria. Furthermore, any other diagnoses must be ruled out before referring an MS patient to a neurologist.

*Early detection and changing diagnostic criteria*

The evolving standards have significantly increased sensitivity, assisting in early diagnosis; nevertheless, specificity must be addressed. Clinical features are, therefore, critical when making an MS diagnosis. It will be easier to diagnose MS early, according to the 2016 MAGNIMS changes to the 2010 McDonald criteria for MRI [283].

*Increased Education and Awareness*

One of the essential variables in early MS diagnosis is raising public and healthcare professionals, awareness and education about the disease. Targeted advertising, workshops, and training programs for primary care physicians and specialists can help achieve this.

*Collaborative Investigation*

Collaboration between academic institutions, government agencies, and private companies can aid in identifying risk factors for MS and developing successful screening programs. This can lead to an early diagnosis and better patient outcomes.

Early MS diagnosis in India is critical for optimal care and improved results. We can enhance MS diagnosis and management in India by raising awareness, implementing current diagnostic procedures, discovering biomarkers, harnessing telemedicine, and encouraging collaborative research.

### 7.2. For the Treatment of MS

Patients could not receive the finest care in the past owing to the scarcity of MS therapy; however, there are now a variety of therapeutic options, but cost remains an issue. According to the expert panel, clinicians should focus on the “3A’s” (affordability, accessibility, and availability) when managing MS. It was suggested that the therapy be made more inexpensive and accessible to patients. One critical factor is the importance of pharmacoeconomic research in MS.

*Priority will be given to counseling*

Patients usually wonder how long their treatment will last. Patients’ concerns may grow if their doctors tell them how long their treatment will last. Counseling is essential for dealing with and controlling the physical and emotional elements of the disease. Furthermore, it assists a patient in starting and sticking with treatment, preventing the development of disability.

Although injectable and oral treatment agents are now widely available, the prescription should be written with long-term efficacy and safety in mind, as well asthe AE profile of the DMDs. Furthermore, the patient should choose the best course of action. Tolerance to therapy needs techniques such as dose titration and reduction, injection timings, use of sleeping aids, co-administration of nonsteroidal anti-inflammatory drugs or acetaminophen (before and after injection), and injection procedures. Interferon is the first-line treatment. If the disease progresses, DMTs are used in the second and third lines of therapy. When relapses occur, patients are given DMT first, followed by methylprednisolone.

*Employment of improved imaging techniques*

Magnetic resonance imaging (MRI) is the most commonly used MSdiagnostic method. Emerging imaging techniques like optical coherence tomography (OCT) and positron emission tomography (PET) may reveal more detailed brain and spinal cord information. These approaches may detect early brain and spinal cord alterations associated with MS, even before symptoms arise.

Machine learning and artificial intelligence (AI) are also being investigated as potential early MS diagnostic methods. AI systems may find patterns and signs related to MS by analyzing huge volumes of patient data, enabling earlier identification and diagnosis. Overall, there are reasons to be hopeful about the future of early MS detection. As technology and medical research develop, we may soon be able to detect MS early, resulting in better patient outcomes.

*Developing new disease-modifying therapies*

Several new therapies for MS are being developed, including ocrelizumab, siponimod, and ofatumumab. These medicines showed promising benefits in clinical trials, and their availability in India may enhance MS management.

*Infrastructure expansion in healthcare*

The Ayushman Bharat scheme, which aims to provide healthcare coverage to more than 100 million families, is one of the efforts made by the Indian government to strengthen the country’s healthcare system. As a result, MS patients in India may now have better access to treatment.

In conclusion, while there are various challenges tomanaging MS in India, several positive advances in MS treatment may benefit patients in the country. The outlook for MS patients in India may improve in the following years as more quality knowledge, access to appropriate healthcare facilities, and advancements in treatment options become available.

## 8. Conclusions

This study examined the difficulties in establishing specific diagnostic biomarkers and treatments for MS in India. We thoroughly covered the different obstacles associated with MS diagnosis and therapy. We identified that biomarkers such as MBP, alpha-antichymotrypsin, anti-mog, miRNA, and others could be future biomarkers for treating and preventing the increased MS disease. There are various challenges todiagnosing and treating MS in India, including a lack of understanding, high prices, and a shortage of specialists. Addressing these difficulties and providing accessible and affordable care for MS patients in India would require a concerted effort from healthcare experts, decision-makers, and the general public.

## Data Availability

Data sharing is not applicable to this article as no datasets were generated or analyzed during the current study.

## Abbreviations

AQP4	Aquaporin-4
CXCL13	C-X-C Motif chemokine ligament
CHI3L1	Chitinase-3-like-1
CDMS	Clinically definitive MS
CIS	Clinically isolated syndrome
COI	Cost of illness
DMT	Disease-modifying therapy
DMA	Disease-modifying agents
DMDs	Disease-modifying drugs
DILI	Drug-induced liver damage
EDSS	Expanded disability status scale
EMA	European medicine agency
EBA	Epstein-Barr virus
GDP	Gross domestic product
GFAB	Glial Fibrillary acidic protein
GA	Glatiramer acetate
HLA	Human leukocyte antigen
HHV-6	Human herpes virus-6
ILs	Interleukins
IFN-γ	Interferon-gamma
IRDA	Insurance regulatory anddevelopment authority of India
iNOS	Inducible-type nitric oxide
IF	Intermediate filament
IBD	Inflammatory bowel disease
MS	Multiple sclerosis
MRI	Magnetic resonance imaging
MHC	Major histocompatibility complex
MOG	Myelin oligodendrocytes
MAGNIMS	Magnetic resonance imaging in MS
NEDA	No evidence of disease activity
NMOSD	Neuromyelitisoptica spectrum disease
NMO	Neuromyelitis optica
NR	Neurology resident
NO	Nitric oxide
NFL	Neurofilament light chain
ONTT	Optic neuritis
OCB	Oligoclonal bands
OCGB	Patched
ON	Optic neuritis
OCT	Optical coherence tomography
PPMS	Primary progressive MS
PET	positron emission tomography
RRMS	Relapsing-remitting MS
RIS	Radiologically isolated syndrome
SPMS	Secondary progressive MS
SNPs	Single-nucleotide polymorphism
TNF-α	Tumor necrosis factor-alpha
Th	T-helper cell
WHO	World Health Organization
